# In vitro hyperspectral analysis of tattoo dyes

**DOI:** 10.1111/srt.13268

**Published:** 2023-01-11

**Authors:** Anna Stolecka‐Warzecha, Łukasz Chmielewski, Sławomir Wilczyński, Robert Koprowski

**Affiliations:** ^1^ Department of Basic Biomedical Science, Faculty of Pharmaceutical Sciences in Sosnowiec Medical University of Silesia in Katowice Sosnowiec Poland; ^2^ Department of Motion Organ Reconstruction Surgery Provincial Specialist Hospital Megrez Tychy Poland; ^3^ Institute of Biomedical Engineering Faculty of Science and Technology University of Silesia in Katowice Sosnowiec Poland

**Keywords:** hyperspectral analysis, radiation reflectance, tattoo, tattoo dye, tattoo removal

## Abstract

**Background:**

There is no method that can guarantee effective, quick, and noninvasive removal of tattoo dyes. Laser methods are considered to be the method of choice. In this study, an attempt was made to determine the in vitro spectral characteristics of selected dyes used in permanent makeup and tattoos and to analyze the obtained parameters in terms of laser treatments optimization.

**Materials and methods:**

Hyperspectral analysis was performed to determine the spectral characteristics of the dye on the entire surface of the slide. Seven dyes used in permanent makeup and tattoos were analyzed in vitro. The maximum reflectance and the wavelength for a given dye were determined for the maximum reflectance in the studied wavelength range: 400–1000 nm. The optical properties of the dyes were determined based on visible light imaging using camera.

**Results:**

The maximum radiation reflectance ranges from 634 to 732 nm for the tested dyes. Visually very similar colors may differ significantly in the wavelength for which the maximum absorption of the radiation occurs. White and yellow dyes are characterized by the highest reflectance value. The black dye is characterized by the lowest reflectance coefficient. Low reflectance of black dye results in more safe and effective removal treatments.

**Conclusion:**

The homogeneity of radiation absorption can be identified using methods of analysis and processing of images in visible light. Optimization of the wavelength of which the maximum absorption/reflectance of radiation occurs may allow us to increase the effectiveness of laser treatments for removing permanent makeup and tattoos.

## 1 INTRODUCTION

A tattoo is an increasingly popular form of body decoration, which bases on implementing dyes to the skin. Originally, the tattoo had a ritual meaning, but now its role is mainly comes down to a decorative function. It is estimated that about 40% of Europeans have at least one tattoo.^1^, ^2^, ^3^ The popularity of tattoos is followed by the need to develop effective methods of removing them. Currently, there is no method that can guarantee effective, quick, and noninvasive removal of tattoo dyes. Nevertheless, laser methods are considered to be the method of choice. The mechanism of interaction of laser radiation with the tissue, which is the basis for the use of lasers in medicine, is based on the theory of selective photothermolysis.^4^, ^5^ Identification of spectral parameters of chromophores (responsible for the absorption of laser radiation) and their distribution is a key factor determining the optimal course of the laser treatment. The currently used methodology for performing laser treatments provides for a priori determination of which chromophore will absorb laser radiation, whereas the type, number, and distribution of chromophores, and even more so their spectral characteristics, are not subject to any objective measurement. As a result, a number of laser procedures do not show the expected effectiveness and/or lead to the occurrence of side effects.^6^, ^7^

Therefore, in this study, an attempt was made to determine the in vitro spectral characteristics of selected dyes used in permanent makeup and tattoos and to analyze the obtained parameters in terms of laser treatments optimization.

### 1.1 Histopathological aspects of dyes implantation into the skin

After implantation, tattoo dyes are deposited primarily in fibroblasts and macrophages of the dermis. Dye‐containing cells are often accompanied by fibrosis. Small amounts of dye are also found in the connective tissue in the form of extracellular aggregates.^8^ The size of the dye particles after implantation into the skin varies between 0.5 and 4 μm. Turquoise and red dyes can create aggregates even twice as large.^9^ The location of the dye varies significantly along with the time passing since implantation. The most important ingredient related to the function of the dyes is the color‐imparting pigment. Both organic and inorganic components are used as pigments. Taking into account the chemical composition, the main elements that build dyes used in tattoos are as follows: aluminum (87%), oxygen (73%), titanium (67%), and carbon (67%). The composition is important for the tattoo removal procedures.^10^

### 1.2 Tattoo removal

Along with the growing tendency of desire to have a tattoo, there is a need to develop effective methods of removing it. It is estimated that about 5% of tattooed people regret their decision and wish to have their tattoos removed.^11^

Among the methods of tattoo removal, some can be mentioned.^12^, ^13^, ^14^

1. Dermabrasion,
2. chemical methods,
3. surgical methods,
4. laser methods.

Laser methods are the method of choice for tattoo removal. They allow remove the tattoo with the highest probability, minimizing the risk of side effects.

### 1.3 Laser tattoo removal

The mechanism of laser tattoo removal is based on the phenomenon of selective photothermolysis. The theory of selective photothermolysis was developed by Anderson and Parish, and it is the base of all currently used laser treatments in aesthetic medicine and dermatology.^15^, ^16^, ^17^, ^18^ According to the selective photothermolysis theory, it is assumed that the tattoo dye is an egosogenic chromophore. Therefore, the laser radiation length used should be selectively absorbed by the tattoo dye. The selectivity of laser radiation absorption is a necessary factor for the laser tattoo removal treatment to be both safe and effective. It should be taken into account that, apart from the tattoo dye, laser radiation can also be absorbed by other naturally occurring chromophores in the skin, mainly water, hemoglobin, and melanin.^17^, ^18^ Absorption of laser radiation by any of the endogenous chromophores (water, melanin, and hemoglobin) warms the skin and consequently causes its thermal damage. Therefore, the length of the laser radiation should be selected in a way, that is, efficiently absorbed by the dye and, as little as possible, by the skin.^15^, ^16^ When optimizing the parameters of the laser radiation in relation to the dye, other parameters should also be taken into account. A very important factor is the depth of laser radiation penetration into the skin. It can be written, to some extent, that the depth of penetration of the laser radiation into the skin is directly proportional to the wavelength of the laser radiation: the longer the wavelength, the deeper the radiation penetrates the skin. Due to the fact that the tattoo dyes are located relatively deep, at the dermo‐epidermal junction, lasers with a wavelength shorter than 500 nm are not useful to remove them. Another key laser performance parameter that should be considered in laser tattoo removal procedures is the pulse duration.^15^, ^17^ The idea behind laser tattoo removal is to supply the tattoo particles with high enough energy to be chemically degraded and shredded. At the same time, it is necessary to be careful not to degrade the skin cells that are in the vicinity of the tattoo particles. In order to be able to choose such a selective action only in relation to dyes and to spare skin cells, it is necessary to use the shortest possible laser pulses. Thus, to remove tattoos, lasers emitting radiation with nanosecond pulse ranges, the so‐called Q‐switched lasers, are used. At the same time, picosecond lasers are becoming more and more popular. In this case, the pulse duration is 10^−12^ s.^17^, ^18^

The mechanism of interaction of laser radiation with dye particles has not been well understood so far. Under the influence of the laser, the optical properties of the dye change. As a result, the pigment discoloration occurs—the spectral range of light absorption in the range of visible radiation changes. The degradation of the dye is done probably by thermal, photochemical, and photoacoustic action, and its fragmentation helps in its removal by the skin's immune system.^15^, ^16^ The effectiveness of laser tattoo removal mainly depends on the following factors: the color of the dye, the chemical properties of the dye, the phototype of the patient's skin, the laser used, the ability to optimize the treatment parameters in relation to the patient, and the ability to perform the procedure correctly.^19^, ^20^, ^21^ At the same time, different dye colors have different absorption maxima, which results in the necessity to use lasers with several wavelengths. Table 1 shows the optimal length of laser radiation (optimal laser) in relation to different dye colors.^22^

**TABLE 1:** Optimum laser types for the removal of particular dye colors23

Laser	Black	Blue	Green	Red
Alexandrite laser 755 nm	X	X	XX	
Ruby laser 694 nm	X	X	X	
Nd:YAG 1064 nm	X	X		
Nd:YAG KTP 532 nm				X

Effective tattoo removal usually requires from 8 to 16 treatments. In the case of multicolored tattoos, it is necessary to perform more treatments—even 20. Considering that the treatments are performed with a 4–6‐week interval, laser removal of the tattoo may last several months. At the same time, when removing color tattoos, several different types of lasers should be used: for example, the Nd:YAG KTP laser for the removal of red dyes, the alexandrite laser for green dyes, and the Nd:YAG laser for black and navy blue dyes.^21^, ^24^ Moreover, dyes are often a mixture of various organic and inorganic pigments, which also makes it difficult to determine their spectral parameters (absorption maxima).^25^

### 1.4 Aim of the study

The aim of this study was an attempt to determine the spectral properties of dyes to maximize the effect of laser radiation absorption by pigments.

## 2 MATERIALS AND METHODS

**FIGURE 1:**
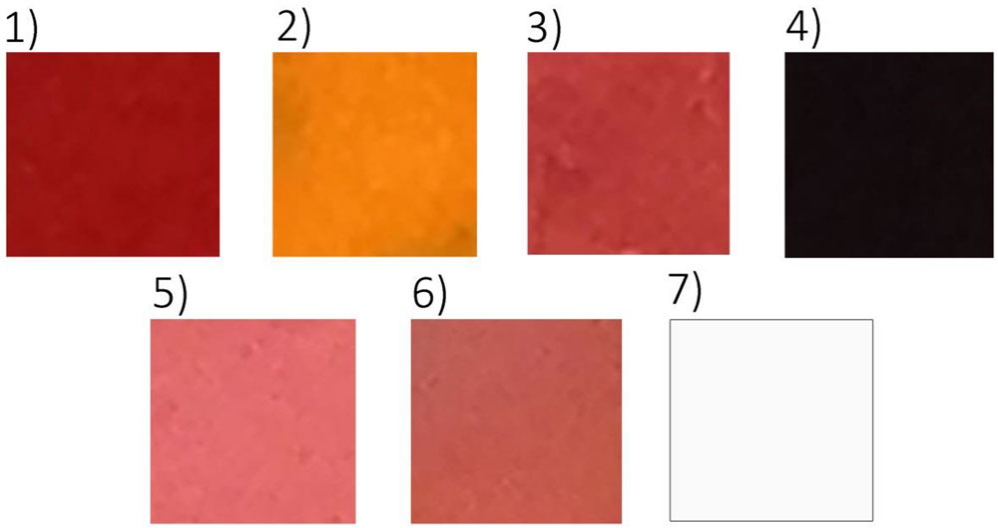
Summary of the dyes used in this study. For dye 7, a black envelope was used.

Seven dyes used in permanent makeup and tattoos were analyzed in vitro (Figure 1). The names of the dyes together with the manufacturer are presented in Table 2. The choice of the above dyes was dictated by the fact that they are especially difficult to remove using laser methods.

**TABLE 2:** List of analyzed dyes

Sample no.	Manufacturer	Manufacturer code	Color
1	Intenze Ink	Sangria	Red
2	Intenze Ink	Rubber doll	Yellow
3	Intenze Ink	Groomy	Dark pink
4	Intenze Ink	Black	Black
5	Intenze Ink	Color pink	Light pink
6	Atomic Ink	Brownish	Brown
7	Hao Tatuaż	White	White

In the first stage of the research, two drops of the dye were placed on the glass slide. Then, using a dry brush, the dye was gently spread over the entire surface of the slide to cover its entire surface. So, prepared slide was left to dry.

In the next stage, the hyperspectral analysis and the analysis in visible light using a classic camera were carried out.

Hyperspectral analysis was performed to determine the spectral characteristics of the dye on the entire surface of the slide. The maximum reflectance and the wavelength for a given dye were determined for the maximum reflectance in the studied wavelength range: 400–1000 nm. The choice of such a wavelength range was dictated by the fact that this range coincides with the wavelengths of lasers used in laser tattoo removal treatments.

In the next stage, the optical properties of the dyes were determined based on visible light imaging using camera.

### 2.1 Hyperspectral parameters analysis

The hyperspectral analysis of the dyes was performed with a hyperspectral camera using image analysis and processing methods.

Computer‐assisted optical imaging is increasingly used in medicine. It allows us not only to compare the features of objects with the pattern but also allows for the biometric assessment of tissue parameters, which is not possible with a standard physical examination.

Hyperspectral imaging is a combination of two noninvasive techniques: imaging and spectroscopy. This allows us to determine both optical and spectral properties of the analyzed objects.

**FIGURE 2:**
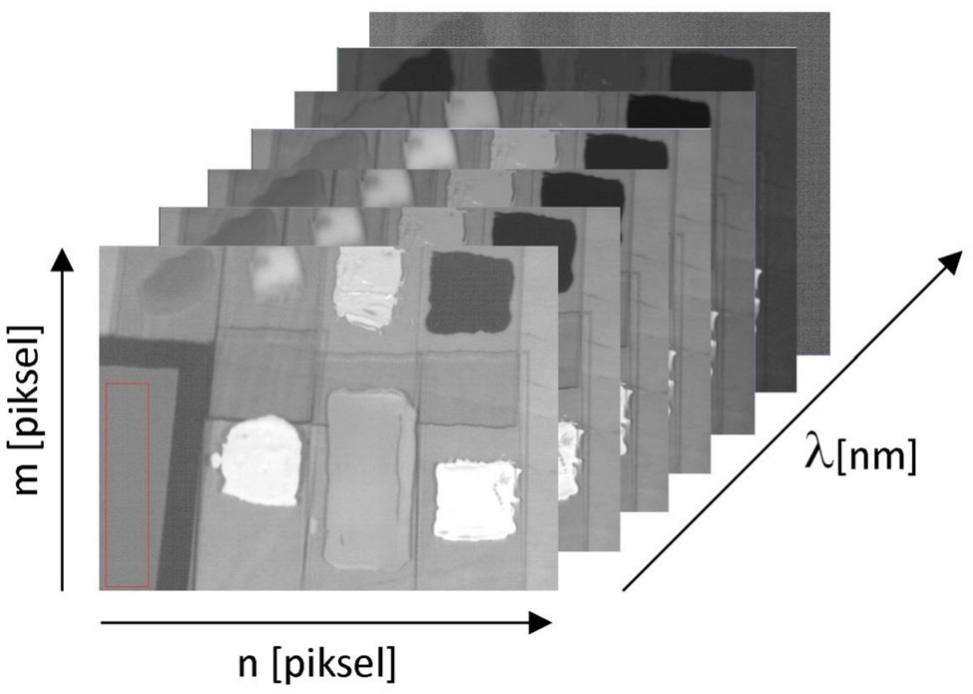
Schematic presentation of the idea of hyperspectral imaging. *m*, *n*, respectively, rows and lines of images, *λ* imaging wavelength range

The hyperspectral camera performs image acquisition by registering radiation with a specific energy intensity (*I*) in a specific range of spatial coordinates (*m*, *n*) and wavelength (*λ*) (Figure 2).

The values of Δ*x* and Δ*y* determine the spatial resolution of an image, whereas the value Δ*λ* determines the spectral resolution. As a result, we receive a series of images of the same object, each recorded at a different wavelengths. This allows us to perform a complex analysis of the optical spectra of the analyzed object as well as the analysis of the entire object by methods of image analysis and processing in a wide spectral range. This allows for material identification (including tissue), anomaly detection, determination of spatial distribution, or target detection.

The molecular hyperspectral imaging is a technique where each pixel is additionally analyzed, so it contains a complete spectral signature in the entire spectral range. So that, it is possible to obtain not only a spatial image of the object, but also spectral information, which is extremely useful in biochemical analysis.

The use of traditional imaging allows you to capture images that are most often displayed and analyzed as an overlay of three channels: Red R, Green G, and Blue B, so the color of the analyzed objects (pixels) depends on the type of incident light. Thus, simple methods of statistical analysis and methods of image analysis and processing do not work well. In hyperspectral imaging, the color is represented by the radiation reflection vector over a wide range of wavelengths. This gives much more information than the three‐component RGB system and allows for multifaceted and multi‐perspective analysis of the assessed objects. A certain disadvantage of hyperspectral imaging is only a small depth of imaging, which results from the scattering of photons on the surface of the object. The average depth of penetration—depending on the wavelength—the longer the wave, the deeper it penetrates—amounts to several hundred microns. Nevertheless, in the analyzed case, it is not an obstacle due to the small thickness of the examined object.

**FIGURE 3:**
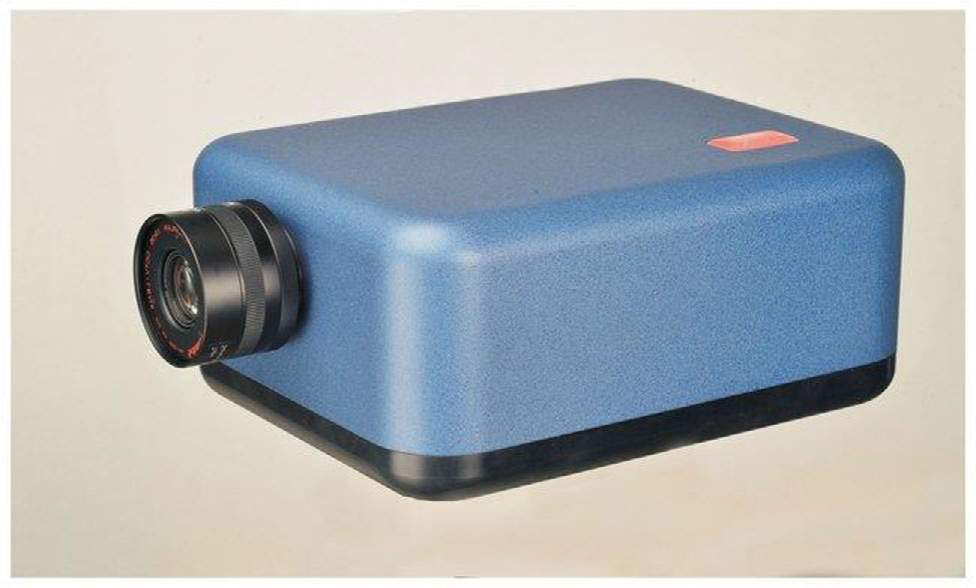
Hyperspectral camera SOC 710, Surface Optics Corporation, San Diego, CA, USA

The spectral parameters of the presented dyes were investigated using the hyperspectral imaging technique. A hyperspectral camera from Surface Optics Corporation, San Diego, CA, USA working in the spectral range of 400–1000 nm was used (Figure 3). The technical characteristics of the camera are presented in Table 3. The spectral range of the camera, for which the optical parameters of the dyes were identified, coincides with the wavelength of the lasers used in laser tattoo removal and permanent makeup treatments.

**TABLE 3:** The technical characteristics of the hyperspectral camera

Spectral resolution	4.69 nm
Spatial resolution	696 pixels per line
Wavelength range	400–1000 nm
Speed of data acquisition	23.2 s per cube
Dynamic range	12 bits
Number of spectral channels	128

Hyperspectral data were obtained by illuminating the tested samples with incandescent light with flat spectral characteristics in the studied wavelength range. Data were recorded with the reflectance standard in the * .cube output format. Then, the acquired data were calibrated according to the registered reflectance standard. The so‐called a gray panel with an identified reflectance range equal to 18% was used. This made it possible to identify the absolute reflectance (absorption) for each of the dyes tested.

The next stage of the research was the identification of spectral parameters of the tested dyes. For this purpose, the MATLAB environment was used created especially for this purpose by Ph.D. Eng. Robert Koprowski from the Department of Biomedical Computer Systems at the University of Silesia in Katowice.

For each of the dyes, the maximum reflectance in the range of 400–1000 nm was determined, for the entire surface of the tested dye.

### 2.2 Analysis of visible light

The analysis of visible light was aimed to identify parameters such as homogeneity and contrast for the entire surface of the tested dyes distributed on the surface of the slide. Due to the fact that after drying the dyes did not have a homogeneous color, it was decided to quantify the color changes for the entire surface of the tested dyes.

For this purpose, the dyes after drying on the slides were photographed using a Canon 5D camera with a Tamron 28–75 mm, f/2.8 USM lens. The tested dyes were illuminated with an incandescent light source with a high color rendering index. The photos were acquired in the * .RAW format.

Then, on each photo of the dyes, a region of interest (ROI) was identified that corresponded to the surface of the dried dye. The prepared images were imported to the MATLAB environment.

The following image parameters were determined:

1. homogeneity,
2. GLCM contrast (Gray Level Co‐occurrence Matrix).

In order to determine and compare homogeneity and contrast among dyes, all images were normalized. Due to the fact that the acquired images were colored, the first step was to transform the color images into gray images. To do this, first the image pixel of a specific color was read with the appropriate share of each of the basic components, and the R, G, and B components were separated. Then the brightness of each of the primary colors was summed, and this sum was divided by 3, without the rest. Then the images were normalized by expanding the range of colors to the full range of grays from white to black (0–255). Contrast and homogeneity were identified for prepared images (normalized in gray scale).

The GLCM method was used to determine the difference among adjacent temperature fields. The application of the GLCM method is designed to determine the homogeneity of the distribution of temperature fields on the thighs of patients before and after the therapy. As a measure of homogeneity, the contrast calculated based on the GLCM matrix was adopted.

The idea of the GLCM method is to calculate in the ROI indicated by the operator or for the whole image, the number of neighborhoods of individual pixels. These neighborhoods can be analyzed from different directions: horizontal, vertical, and diagonal. The closest possible neighborhoods are analyzed most often—for example, horizontally these are two pixels adjacent to each other. The number of neighborhoods between each pixel brightness is recorded in the GLCM by specifying the number of neighborhoods between pixels “1” and “0.” The contrast is then calculated based on the GLCM matrix.

In the example described above, a two‐dimensional model (0,1) was used, whereas in the analyzed case, an *n*‐dimensional model was used, where *n* is the brightness difference between neighborhood image pixels. The GLCM analysis in this case included pixels adjacent to each other on the right side in the lines of the rows.

The homogeneity test was aimed at determining the brightness uniformity across the entire ROI. In the adopted research model, homogeneity is understood as

$$\sum_{i,j}\frac{p\left(i,j\right)}{1+\left|i-j\right|}$$

where *i* is the brightness of the tested pixel; *j* is the brightness of the adjacent pixel.

## 3 RESULTS

### 3.1 Hyperspectral analysis

The proposed method of hyperspectral analysis allowed us to obtain a series of images recorded at successive wavelengths. A total of 128 images were recorded.

**FIGURE 4:**
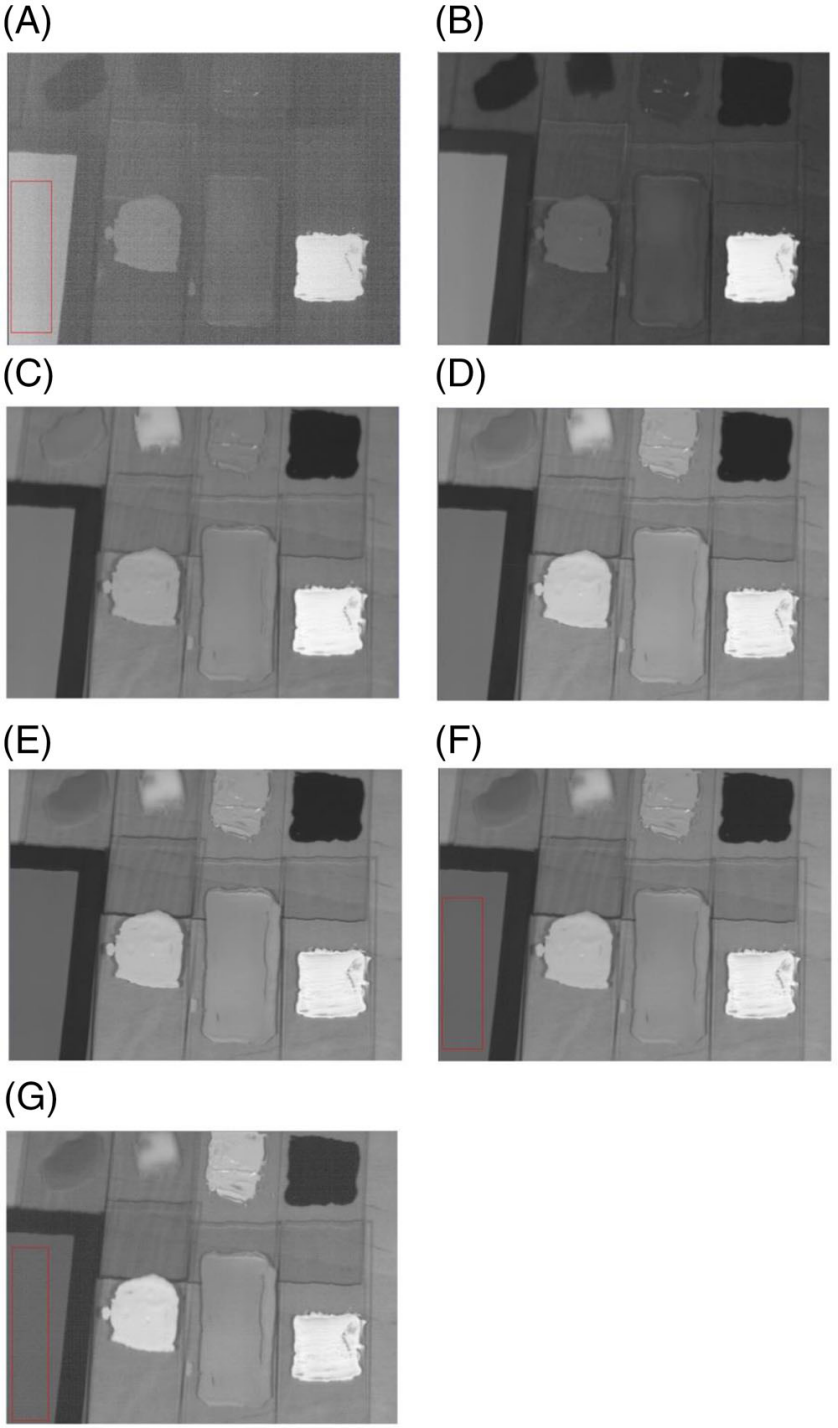
Examples of photos recorded at the following wavelengths along with fragment of gray panel (standard): 420.98 nm (A), 501.84 nm (B), 599.31 nm (C), 703.59 nm (D), 798.73 nm (E), 901.08 nm (F), and 999.30 nm (G)

Examples of photos recorded at the following wavelengths are shown in Figure 4: 420.98 nm (A), 501.84 nm (B), 599, 31 nm (C), 703.59 nm (D), 798.73 nm (E), 901.08 nm (F), and 999.30 nm (G).

Due to the fact that the presented images have been calibrated according to the standard (the gray panel shown in Figure 4 with reflectance of 18%), they are presented in gray. The brightness of the image is a measure of reflectance in the adopted model: the brighter the image, the higher the reflectance. Thus, a white pixel (RGB 255, 255, 255) represents to a reflectance of 100% (1.0) and a black pixel (RGB 0.0.0) a reflectance of 0% (0.0).

**FIGURE 5:**
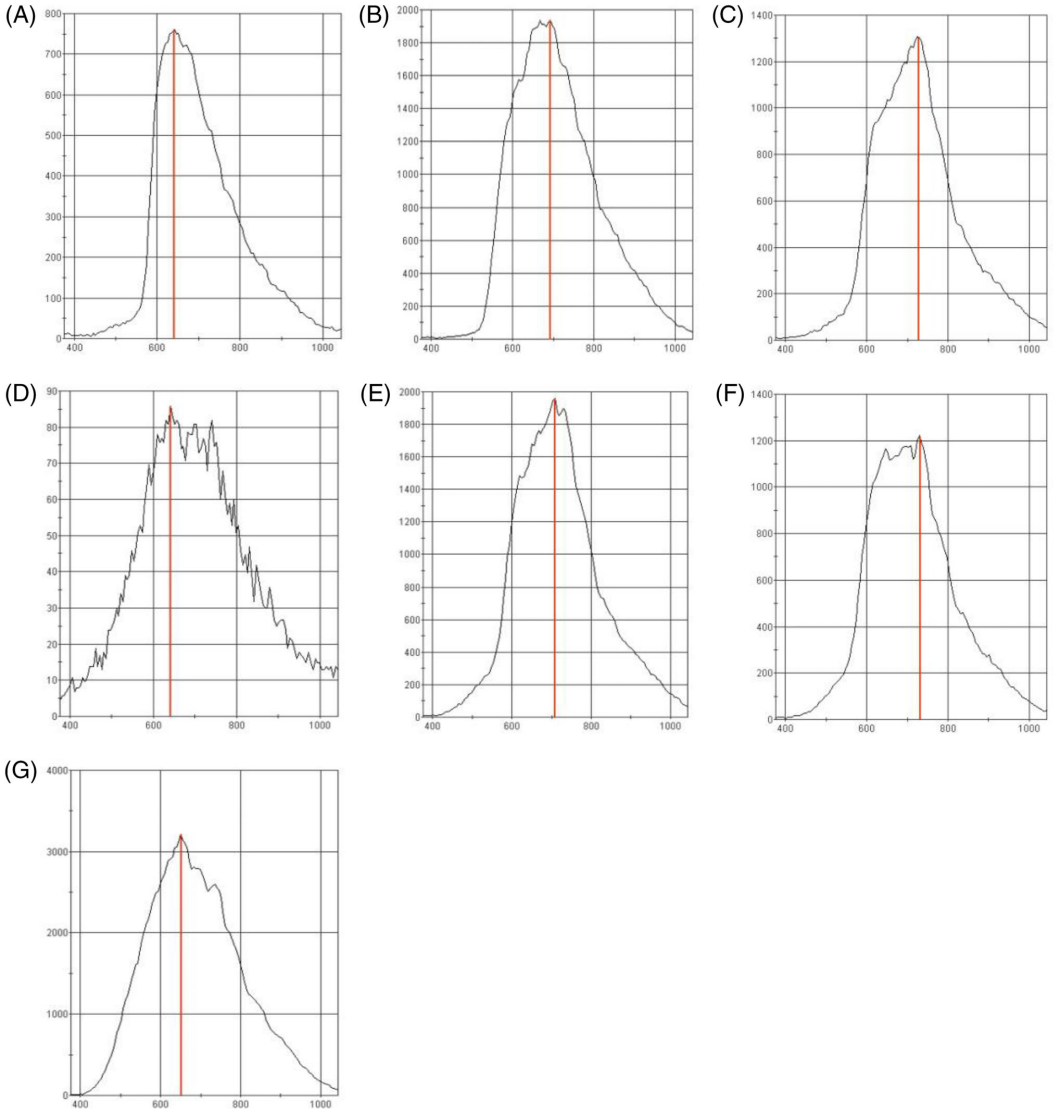
Reflectance spectra of all registered dyes respectively: red (A), yellow (B), dark pink (C), black (D), light pink (E), brown (F), and white (G)

Figure 5 shows the reflectance spectra of all tested dyes. The wavelength (nm) is marked on the *x*‐axis and the reflectance measured in relative units on the *y*‐axis. All dyes were registered in one session to eliminate the risk of uneven lighting of the samples or the influence of other factors.

**FIGURE 6:**
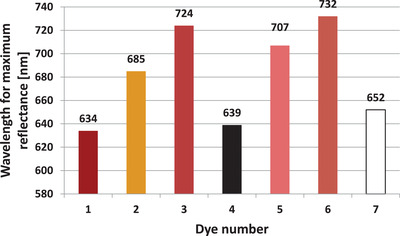
Wavelength of all tested dyes for which the maximum reflectance was identified

Figure 6 shows the wavelength of dyes for which the maximum reflectance of radiation occurs in the range of 400–1000 nm. There is a slight discrepancy between the obtained reflectance maxima: The lowest wavelength for which the maximum reflectance occurs is the red dye (No. 1) (634 nm), whereas the dye for which the highest wavelength was identified at the maximum reflectance is the brown dye (No. 6)—732 nm.

**FIGURE 7:**
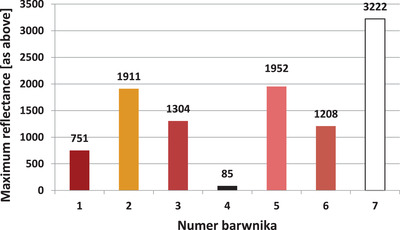
Maximum reflectance for all dyes tested identified in the wavelength range from 400 to 1000 nm

The maximum reflectance for each dye is shown in Figure 7. The reflectance in implemented research model is understood as the power ratio of the reflected beam to the power of the incident beam on the border between two mediums with different refractive indexes.

It should be emphasized that there are significant differences in the maximum reflectance values. And so, for the black color (dye no. 4), the maximum reflectance is 85 relative units. In turn, for the white pigment it is almost 38 times higher and amounts to 3222 relative units.

The relatively large difference in reflectance between similar colors is also noteworthy. And so, for the dye no. 1 (red), reflectance is 751 relative units, and for dye no. 3 (dark pink), it is almost twice as high: 1304 relative units.

It should be noted that reflectance, in addition to the optimal wavelength of laser radiation, is a factor determining the effectiveness of laser tattoo removal treatments. According to the assumption that reflectance is inversely proportional to absorbance (with little influence of transmittance), it can be assumed that high reflectance will reduce the effectiveness of laser tattoo removal treatments. Thus, based on the results presented in Figure 7, it can be concluded that black dye will be the easiest to remove, and then in descending order: brown, red, dark pink, yellow, light pink, and white. In the case of the white dye, almost all of the incident energy is reflected and/or dissipated. The dye will therefore not absorb the laser radiation and will be extremely difficult to remove.

### 3.2 Analysis in visible light

In addition to the hyperspectral analysis, a visible light analysis was also performed. This analysis was aimed to determine the homogeneity of the distribution of chromophores inside the dyes. For this purpose, the GLCM contrast of the tested dyes and their homogeneity were determined.

**FIGURE 8:**
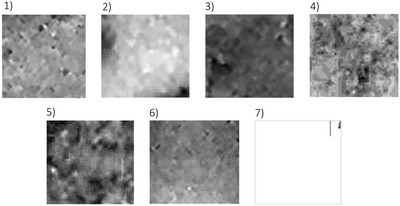
Normalized images of test dyes recorded in visible light. An envelope was used for dye no 7.

Images of the analyzed dyes in visible light after performing normalization procedure are shown in Figure 8. The purpose of the normalization was to make it possible to compare the brightness of individual pixels between the tested dyes. The normalization was carried out for each of the images recorded in the RGB format (Figure 1), and the brightness was extended to the full range from 0 to 255. Thus, in the image of each dye, the brightest pixel was identified and was given the brightness of 255 (white) and the darkest pixel the brightness of 0 (black). The brightness of the remaining pixels was then extended proportionally over the full range of gray levels.

**FIGURE 9:**
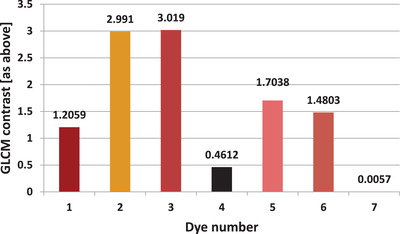
Gray Level Co‐occurrence Matrix (GLCM) contrast of all registered dyes determined based on photos recorded in visible light

**FIGURE 10:**
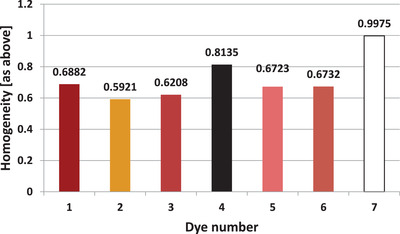
Homogeneity of all registered dyes determined based on photos recorded in visible light

For the dye images shown in Figure 8, GLCM contrast and homogeneity were calculated. The determined parameters of GLCM contrast and homogeneity are presented in Figures 9 and 10, respectively.

The large discrepancy in the results between the individual dyes draws the attention. Moreover, there is a clear negative correlation between the calculated parameters of the image analysis in the visible light. The higher the contrast, the lower the homogeneity. The Pearson correlation coefficient over the entire range of dyes measured for these two parameters is −0.8835.

The dark pink dye (No. 3) is characterized by highest contrast (and at the same time one of the lowest homogeneities), respectively: 3.019 and 0.6208. On the other hand, white dye is characterized by the highest homogeneity and the lowest contrast, respectively: 0.0057 and 0.9975.

## 4 DISCUSSION

The method of choice for tattoo removal is laser techniques. During the procedure, laser radiation should be selectively absorbed by the dye. Under the influence of the absorbed laser radiation, the dye is fragmented, which facilitates its removal through macrophages and conditions changes in the optical parameters of the dye, which results in a reduction of radiation absorption in the range of visible radiation.

However, it should be emphasized that the current laser tattoo removal methods are not fully effective and can cause serious side effects. In a study on 20 patients, Cannarozzo et al.^26^ showed that QS Nd:YAG laser may be considered a good choice in the treatment of cosmetic tattoos, because it enables complete removal with acceptable secondary effects. In this case, however, the permanent makeup of lips and eyebrows were removed, small in terms of area and color diversity.

Side effects are most often the result of insufficient absorption of laser radiation by the tattoo dye and intense absorption of radiation by endogenous skin chromophores: mainly melanin and hemoglobin. As a result of tattoo removal, there are also unusual complications, such as crescent‐shaped incisional cuts, described in the manuscript of Agrawal et al.^27^

Klein et al. presented the results of a survey conducted on 157 respondents and indicated that one third of participants were unsatisfied with the result of laser tattoo removal, and a complete removal of the tattoo pigment was obtained in 38% only. Local transient side effects occurred in nearly all participants, but an important rate of slightly visible scars (24%) or even important scarring (8%) was reported.^28^

Accordingly, new solutions are sought that would help increase the effectiveness of laser tattoo removal treatments while minimizing side effects. No studies have been published that could indicate the objectification of parameters in relation to specific dyes verified by hyperspectral methods so far.

In this study, an attempt was made to determine the spectral parameters of dyes, which are particularly resistant to laser removal treatments. The wavelength of which the maximum reflectance of the laser radiation occurs was determined using hyperspectral imaging. The tested radiation ranged from 400 to 1000 nm, which covers almost 100% of currently used non‐ablative lasers areas.

The obtained results show that for the whole group of dyes tested, lasers with emission ranging from 634 to 732 nm should not be used. A hyperspectral profile was determined for each dye, which allowed us to optimize the laser radiation length. It is worth emphasizing that potentially similar dyes, such as light pink and dark pink, have significantly different spectral profiles. Thus, it is not possible to determine the optimal wavelength of the laser radiation used for its removal solely based on the color of the dye. These significant differences in spectral profiles probably results from the mixture of numerous compounds in a dye, causing a specific shade of color. Even a small (quantitatively) admixture of another pigment can significantly affect the spectral parameters.

Moreover, the maximum reflectance was determined for all analyzed dyes. It should be noted here that reflectance, next to the optimal wavelength, will be a key parameter responsible for the effectiveness of laser tattoo removal treatments. It should be expected that for high reflectance pigments—such as white and yellow, their removal from the skin will be extremely difficult and will require at least a dozen or so treatments. On the contrary, for dyes with low reflectance (high absorbance), laser tattoo removal will be much easier.

It is not possible to estimate the reflectance from the color of the dye alone same as with the optimal wavelength. And so for dyes 1 and 3 (red and dark pink, respectively), the brightness is similar, whereas the reflectance is almost twice as high in the case of the dark pink dye.

In addition to the hyperspectral analysis, the GLCM contrast and homogeneity for images recorded in visible light were also determined. The aim of the analysis was to determine the homogeneity of the distribution of chromophore molecules within the dyes. The white dye is the most homogeneous and the yellow dye the least homogeneous. In the case of white dye, its chemical composition is probably responsible for its high homogeneity. White dyes are made of micronized titanium dioxide. The use of only one pigment allows the creation of a highly homogeneous suspension.

In yellow dyes, mixtures of many different pigments, including organic and inorganic, are used, which may result in lower homogeneity.

Summing up, it should be stated that the proposed method can find practical application in laser tattoo removal treatments. If the manufacturer verified the spectral properties of the dyes and placed such information on the packaging, it would enable the optimization of laser radiation parameters. This founding is valid only if the spectral parameters remain unchanged during the entire lifetime of the dye.

Moreover, it should be emphasized that the conducted analyzes were performed in the form of in vitro tests. Thus, the spectral parameters of the skin, where the dyes are implanted, were not considered. The optimal solution, but practically very difficult, would be to verify the hyperspectral parameters of the tattoo in vivo, which creates opportunities for further research in this area.

## 5 CONCLUSIONS

The conducted research allows us to present the following conclusions:

1. The maximum radiation reflectance ranges from 634 to 732 nm for the tested dyes.
2. Visually very similar colors (e.g., red and dark pink) may differ significantly in the wavelength for which the maximum absorption of the radiation occurs.
3. White and yellow dyes are characterized by the highest reflectance value. This determines difficulties in elimination them from the skin with laser methods.
4. The black dye is characterized by the lowest reflectance coefficient—almost 40 times lower than the white dye.
5. Low reflectance of black dye results in more safe and effective removal treatments.
6. The homogeneity of radiation absorption can be identified using methods of analysis and processing of images in visible light.
7. Optimization of the wavelength of which the maximum absorption/reflectance of radiation occurs may allow us to increase the effectiveness of laser treatments for removing permanent makeup and tattoos.

## CONFLICT OF INTEREST

The authors declare that there is no conflict of interest that could be perceived as prejudicing the impartiality of the research reported.

## Data Availability

The data that support the findings of this study are available from the corresponding author upon reasonable request.
